# Supplementary material for: Barriers to the uptake of eye health services of the children in rural Bangladesh: A community-based cross-sectional survey
Source: PLoS One. 2023 Dec 7;18(12):e0295341. doi: 10.1371/journal.pone.0295341 (PMC10703229; doi:10.1371/journal.pone.0295341)
Supplement: S2 File — (DOCX) [file pone.0295341.s003.docx]

১ম প্রশ্নকর্তাঃ নিচের প্রশ্ন দেখা যায় ?

উত্তরদাতাঃ জ্বি স্যার ,দেখা যায়। হোয়াট আর দ্য কজেজ এন্ড সিচুয়েশন রিগার্ডিং ডিলে হেলথ সিকিং বিহেভিয়ার অফ প্যারেন্টস ক্যান বি এভার?

১ম প্রশ্নকর্তাঃ বাংলাটা সুস্পষ্ট হইছে আর কি।

উত্তরদাতাঃ হ্যাঁ হ্যাঁ। বেশির ভাগ বাবা মার সন্তানদের চোখের ত্রুটি আছে তারা চোখের ত্রুটি আছে । আপনার কাছে কী কারণে চোখের ত্রুটি নিয়ে ডাক্তারের কাছে যায় না?

আচ্ছা প্রথমত কারন বাচ্চারা কোন কমপ্লেন করতে পারে না এটা প্রথম কারণ। কারণ গত সপ্তাহে একটি বাচ্চা পাইছি টু স্ট্রেস ৫ হয়ে গেছে ডাক্তার দেখাইতে বলছে বাবা মা আসে নাই ঠান্ডার জন্য। যেহেতু বাচ্চাটা কোন কমপ্লেন করতে করতে পারছে না সেহেতু বাবা- মা বুঝতে পারছে না বাচ্চাটা কম দেখতে পাচ্ছে বা চোখে কোন অসুবিধা হচ্ছে। আর চোখের অধিকাংশ ত্রুটি যেগুলো জন্মগত ত্রুটি এতে কোন পেইন থাকে না। বিশেষ করে ক্যাটারেক্ট, এমনকি ইস্কুইন্টও। বাচ্চা যেহেতু কিছু বলতে পারে না, কমপ্লেন করতে পারে না এটা মনে হয় আমার কাছে এক নাম্বার কজ।

আরেক টা ব্যাপার হচ্ছে কি স্যার অপটামজোলজিস্ট দের কাছে পাঠালে মনে করি আচ্ছা ঠিক আছে যাবো বা যাচ্ছি। ঠান্ডা একটা কারণ ইদানিং দেখা যাচ্ছে ছোট বাচ্চা ঠান্ডার মধ্যে কেম্নে নিয়ে আসবো সকাল বেলা। এগুলো একটু ব্যাপারটা মেইন কারন বলে আমার মনে হয় স্যার ।

২য় প্রশ্ন কর্তাঃ স্যার এই ক্ষেত্রে কি কোন ধরনের স্যোসাল নরমস আমরা যেটাকে বলি এটা কি কোন ইফেক্ট ফেলে যে বাচ্চাকে আমরা দেরিতে আনতে চাচ্ছি বা কোন কিছু?

উত্তরদা্তাঃ স্যোশাল নরমস অবশ্যই একটি ব্যাপার কারন বাচ্চাগুলোকে বাইরে আনতে গেলে তারা অনেক সময় বিভিন্ন ধরনের কালো তিল টিপ এগুলো দিয়ে আনে ।অনেকের ধারনা হাসপাতালে দেখাতে নিলে আরো চারপাচটা রোগ হবে । তো অনেক সময় একটু এভয়েড করে না বাচ্চার তো কোন অসুবিধা নাই বিশেষ করে দাদি নানীর আমরা এডুকেশনাল ব্যাকগ্রাউন্ড একটা বড় ফ্যাক্টর হিসেবে কাজ করে। কারণ তারা অনেক সময় তাদের ওয়াইফদেরকে বা ছেলের বউদেরকে বলে না থাক বাচ্চা তো ভালো আছে। হাসপাতালে নিবা আর চারটা রোগ হবে। সুতরাং এক্ষেত্রে যখন আমরা বাচ্চাদেরকে ডিউরিং এক্সামিনেশন করি তখন গাইনাকলোজিস্টরা ডেলিভারির পরেও স্পেসিফিক যদি অবশ্যই একটা শব্দ যুক্ত করে দেয় অবশ্যই বাচ্চাটাকে চোখ দেখাবেন।

স্কুলে ভর্তির আগে অবশ্যই বাচ্চাটাকে চোখের পরীক্ষা করে নিবেন। দেখা যায় বাচ্চাগুলা ফিক্সডলি একটা আই চেক আপ পায়। স্যারের তো একটা বড় প্রজেক্ট আছে ইপিআই এর সাথে যদি একটা বাচ্চাদের চোখ গুলো দেখে দেয় আমাদের ইপি আই টেকনিশিয়ান বা এরা যারা আছে অথবা যদি আমরা এক সময় আমরা আপনার গাইডেন্সে কাজ করছিলাম যেমন-স্কুইড, টোসিস অনেকগুলো প্যাশেন্ট স্যার আমরা পাইছি বাবা- মারা এগুলা নিয়ে বসে থাকতো যেমন- লক্ষ্মীট্যারা যদি থাকে এটা জন্মত্রুটি এটার চিকিৎসা হবে না। তো এগুলার যে চিকিৎসা আছে বিশেষ করে স্কুইড, টোসিস, কুইন্ট বিশেষ করে এই ধরনের জিনিসগুলার যে চিকিৎসা আছে এগুলা যদি আর্লি ডিটেক্ট করা যায় তো স্যার এগুলা মনে হয় স্যার বাচ্চাগুলা আর্লি ইয়া পাবে।

২য় প্রশ্নকর্তাঃ তো সেইক্ষেত্রে কি স্যার জেন্ডারের কোন রোল আছে ?

উত্তরদাতাঃ অব্যশই একটা জেন্ডারের রোল আছে। কারণ আমরা যেটা পাই অধিকাংশই বাবা বিদেশে থাকে হয়তোবা আমরা যেটা দেখতেছি তো মারা দেখা যায় কি বাচ্চাদেরকে নিয়ে শ্বশুরবাড়ির যে বাইন্ডিংস সে বাইন্ডিংস থেকে বের হয়ে আসতে একটু অন্যের উপর ডিপেন্ড করতে চায়। দেখা যায় আমি বলছিলাম ওর বাবাও ফোনে বলছে কিন্তু ওর চাচা সময় দিতে পারতেছে না বা পরে ওর মামাকে নিয়ে আসছি এ ধরনের একটা ব্যাপার। আর মেল বাচ্চাগুলা অবশ্যই প্রায়োরিটি বেশি পায়।।

২য় প্রশ্নকর্তাঃ সেক্ষেত্রে কি মেয়ে ছেলের কোন ডিফারেন্স আপনি কখনো দেখেছেন ?

উত্তরদাতাঃ ওরকম একটা চোখে পড়ে নাই। তবে দেখা যাচ্ছে যে ছেলে বাচ্চা হলে একটা প্রায়োরিটি দিচ্ছে আগে ডাক্তারের কাছে বা ডাক্তারের নোটিশে আসে।

২য় প্রশ্নকর্তাঃ এছাড়া কি আপনি আর কোন কজেস বা সিচুয়েশন পেয়েছেন যে কারণে হেলথ সিকিং বিহেবিয়্যার করতে দেরি হচ্ছে তাদের স্যার ?

উত্তরদাতাঃ হ্যাঁ আমি যদি আই রিলেটেড বলি তাহলে (অস্পষ্ট)। এভেইলিবিলিটি বড় একটা ফ্যাক্টর। এটা ইউনাইটেড থেকে ধরেন সাতকানিয়া চলে গেছে।

৫ মিনিট

এখন দেখা যাচ্ছে যে উপজেলাতে হয়তোবা চোখের ডাক্তার পাচ্ছে না হয়তোবা চিটাগাং এসে দেখাতে হবে বা বড় উপজেলায় যেতে হবে সেজন্য একটু সময়টা নিচ্ছে। না আমি শীত এ আসতে হবে ঠান্ডার মধ্যে বাতাস লাগবে। এজন্য একটু দেরি করে আসতেছে। সেক্ষেত্রে এভেইলেবিলিটি অফ অপটামলজিস্ট একটা বড় একটা ব্যাপার। যেকোন ডাক্তারদের এভেইলএবিলিটি বড় একটা ফ্যাক্টর। না সে হয়তো শহরে বড় ডাক্তার দেখাবে সেজন্য একটু ওয়েট করতেছে। উপজেলায় যাবে সেজন্য একটু ওয়েট করতেছে।

প্রশ্নকর্তাঃ আর কোন ফাইনানশিয়াল কজ কি আছে? যে কারনে প্যারেন্টসরা ডিলে হেলথ সিক করতেছে চোখের ব্যাপারে ?

উত্তরদাতাঃ ড্যাফোনিটলি ড্যাফেনিটলি ড্যাফেনিটলি । ড্যাফিনেটলি ব্যাপারটি যদি আমি এখানে বলি এই যে বাচ্চাদের ট্রান্সপোর্ট আসবে।মেডিকেলে থেকে লিখে দিছে যে ১ মাস বয়সের বাচ্চাদের চোখ পরীক্ষা করাতে হবে। সে চলে গেছে, সে আবার আসবে তার বাবা হয়তো দেশে নাই বা অবস্থা নাই তাই একটা সিএনজি নিয়ে আসতে হবে সেক্ষেত্রে সে হয়তো আরো কিছুদিন ডিলে করতেছে। ফাইনানশিয়াল কজ তো অবশ্যই বাংলাদেশের পারসপেক্টিভে বড় একটা ফ্যাক্টর তার এই ডিলের পিছনে।

২য় প্রশ্নকর্তাঃ কখনও কি প্যারেন্টসরা এসে ডিরেক্ট কি এই ধরনের প্রবলেমগুলা বিশেষ করে ফাইনানশিয়াল প্রবলেমগুলোর কথা এটা কি তারা কখনও মেনশন করে যে হচ্ছে হলো গিয়ে আমাদের ফাইনানশিয়াল কজের কারণে আমরা আসতে পারতেছি না?

উত্তরদাতাঃ না এটা আমি বাচ্চাদের ক্ষেত্রে আমরা পাই না। আমরা ফাইনানশিয়াল পায় বড়দের ক্ষেত্রে যেটা পেলেও আর কি আমরা বলতে চায় না আর কি আমরা আর্থিকভাবে অস্বচ্ছল বা দুর্বল। অনেক সময় আমরা বাবা-মারা স্বীকার করতে চায় না বিশেষ করে সন্তানদের ক্ষেত্রে তো নাইই। অনেক সময় ক্যাটারেক্ট রোগীর ক্ষেত্রে পায় যে আপনি অপারেশন করেন নাই কেন (অস্পষ্ট)। তখন হয়তোবা বলে যে টাকার অভাবে করতে পারি নাই। অনেক আগেই ডাক্তাররা বলেছিল অপারেশন করার জন্য। এখন দেখা যাচ্ছে আমাদের ন্যাশনাল আই কেয়ার আছে তখন দেখা যাচ্ছে ওরা বলছে টাকা লাগবে না এজন্য আসছি। এ ধরনের কিছু প্যাশেন্ট পায় কিন্তু বাচ্চাদের ক্ষেত্রে আসলে বাবা-মা আসলে সরাসরি এসে কিছু বলে না কিন্তু ঐ যে আমরা বুঝি যে সে হয়তো তার ফাইনাশিয়াল কারণে একটু ডিলে করছে বাট সরাসরি আমাদের কাছে কেউ ঐভাবে কেউ অভিযোগ করে নাই।

২য় প্রশ্নকর্তাঃ তাহলে স্যার এই যে প্যারেন্টসরা বা কেয়ার গিভাররা ডিলে হেলথ সিক করতেছে এবং তার ফলে ক্লিনিক্যাল আইয়ের অনেক ধরনের প্রবলেম ডিলে রিপোর্টিং এর কারণে হচ্ছে আর কি। সেক্ষেত্রে আপনার সাজ্রশন্স কি থাকবে স্যার যে এইভাবে আমাদের সিচুয়েশনটাকে ইমপ্রুভ করতে পারি?

উত্তরদাতাঃ স্টং রেফারাল সিস্টেম হচ্ছে বাবা-মা কে বুঝিয়ে বলতে হবে আপনার বাচ্চার এখন ভালো আছে চোখ যখন নিউ বর্ন ডেলিভার হয় তখন আপনার বাচ্চাকে অবশ্যই যদি সে প্রিম্যাচিউর বেবি হয় এক মাস বয়সে চোখটা দেখাতে হবে। অবশ্যই কথাটা উল্লেখ করতে হবে। আর বাচ্চাটা যদি নরমাল বাচ্চা হয় সামথিং আদার কোন এবনররমালিটিস তাহলে আমরা অবশ্যই বলি তার এ জন্মগত রোগ আছে তার চোখের পাতাটা পড়া বা চোখের পাতাটা বাকা আপনাকে অমুক জায়গায় গিয়ে বা বড় ইন্সটিটিউট সেখানে গিয়ে অবশ্যই যাতে সে চোখটা দেখায়। এই অবশ্যই কথাটা বলে গুরুত্ব বুঝিয়ে দিতে হবে। বুঝিয়ে দিতে হবে এই যে ট্যারা চোখ এটা ট্যারা না চিকিৎসা না করলে বাচ্চাটা দেখা যাচ্ছে একটা চোখ লেজি আই এমনকি ব্লাইন্ডের দিকে চলে যায়। বাট টোসিস অবশ্যই গুরুত্ব দিয়ে বুঝিয়ে দিতে হবে। সুতরাং উত্তরণের পদ্ধতি বাবা-মাকে এ বিষয়ে সচেতন করতে হবে। আমরা সবাই ইপিআই টিকা দিই বাবা-মারা জানে আমাদের দেখতে হবে। এরকম একটা প্র্যাক্টিস যদি ডেভলপ করে যে প্রত্যেকটা বাচ্চা স্কুলে যাওয়ার আগে অবশ্যই চোখটা টেস্ট করাবেন। তখন এই জিনিসটা হয়তোবা অনেকটা কমে আসবে। তো স্কুলগুলো যদি বলে তাদের বলে যে না আমার বাচ্চারা ভর্তির আগে একটা চোখের সার্টিফিকেট লাগবে বা আমাদের এই যে ইপি আই টিকার সাথে যদি বাচ্চা প্রিম্যাচিউর হয় ওকে স্ক্যানিং করায়েছেন কিনা এ ধরনের একটা ব্যাপার যদি তারা যদি একটা নোট দেখতে চায় যে না চোখটা স্ক্যানিং করানো হয়েছে চোখটা ভালো ছিল।এখন দেখা যাচ্ছে অনেক বড় বড় সেন্টারগুলা মানে এমনকি জ্বীনগত কোন ত্রুটি আছে কিনা এটাও কাউন্সিলিং করে। এত আমাদের দরকার নাই । তার জন্মগত ত্রুটি আছে কিনা এরকম যদি একটা প্রেডিকশন লিখে দেয় যে জন্মগত ত্রুটি নাই তাহলে চোখটাও চেকের মধ্যে হয়ে গেল। তার স্কুইট, টোসিস, ক্যাটারেক্ট এগুলো আমরা অনেক আর্লি ডিটেক্ট করতে পারবো।

২য় প্রশ্নকর্তাঃ জ্বী স্যার থ্যাংক ইউ।

১০ মিনিট
