# Supplementary material for: Barriers to the uptake of eye health services of the children in rural Bangladesh: A community-based cross-sectional survey
Source: PLoS One. 2023 Dec 7;18(12):e0295341. doi: 10.1371/journal.pone.0295341 (PMC10703229; doi:10.1371/journal.pone.0295341)
Supplement: S3 File — (DOCX) [file pone.0295341.s004.docx]

Well, the first reason is that children can't complain. Because I got a baby last week, Two Stress 5, the doctor says the parents didn't come for the cold. Since the child is unable to make any complaint, the parents are not able to understand that the child is seeing less or there is any problem in the eyes. And most of the eye defects that are birth defects do not have any pain. Especially cataracts, even ascites. Since the child cannot say anything, cannot complain, it seems to me to be the number one cause of delayed health seeking behavior.

Another thing is that if you send child to the optometrists, they think it's okay to go or not to go. One of the reasons for the cold has recently been seen how to bring a small child in the cold in the morning. I think that's one of the main reasons.

Social Norms are a must because they often bring a variety of black mole tips to bring out the kids.Many people think that if they show up at the hospital, they will be affected by four or five more diseases. So many times especially the grandparents' educational background works as a big factor. Because they often tell their wives or sons' wives that the child is fine. So in this case, when we do the examination of the child, the gynecologist must show the eyes to the child even after delivery, if a specific word is added. They can just tell them, make sure your child has an eye examination before going to school. So that the kids get an eye check-up timely. We have a big project with EPI, if a child's eyes are seen by our EPI technician then squint and ptosis can timely diagnosed. Many patients we have found that parents used to sit with them and told that, like – squint is by born, it will not be treated. So the treatments are there for these especially the squint, ptosis, quint. Especially if they can be detected early then these kids will get early and better treatment and outcome is outstanding.

There is definitely a gender role. Because most of what we find is that the father is abroad, maybe what we see is that the father-in-law's bindings with the children want to come out of the bindings and depend on others. Apparently, I was saying that his father is also talking on the phone, but his uncle is not able to give time or I will bring his uncle later. And, of course, the kids get more. There is no difference between a boy and a girl. However, it appears that the boy is giving a priority to the child before the doctor or the doctor's notice comes.

Affordability is a big factor. Now it seems that maybe the eye doctor is not available in the upazila, maybe it is taking a little time to come to main district or to go to the big upazila. No, I'll have to come in the winter. That's a bit late. In that case, the availability of optometrists is a big thing. The availability of any doctor is a big factor. He may be waiting for a big doctor in town. He is waiting a bit to go to the upazila. The doctor has written that the eyes of children up to 1 month of age should be examined. He's gone, he'll come back, his father may or may not be in the country, so he'll have to bring a CNG. Financial reasons are definitely a big factor in Bangladesh's perspective behind this deal. We get financial problem for adults what we get and what we do not want to say and whether we are financially unsound or weak. Sometimes we don't want to acknowledge our parents, especially when it comes to our children. Many times the cataract patient finds out why you did not operate. Maybe it was because I didn't have the money. Doctors had suggested for surgery. Now it looks like we have national eye care, then it looks like they say they don't need money so I'm coming. Some of these patients get it, but in the case of children, the parents don't actually come directly and say anything, but that we understand that he may be a little bit late for his financial reasons, but no one has complained to us directly.

The strong referral system is to explain to the parents that your baby's eyes are fine now, when the new born is delivered, your baby must show his eyes at the age of one month if he is a premature baby. Of course, it should be mentioned. And if the child is a normal child, then we must say that he has this congenital disease, his eyelid is falling or the eyelid is crooked, you must go to some place or to a big institute so that he can see his eyes. This must be explained. It has to be explained that if the squint eye does not treated, the child will see that one eye goes to the lazy eye and even the blind. The butt toss must be explained with importance. Hence, the parents should take good care in this regard. We all get the EPI vaccine, parents know we have to see. If such a practice develops that every child must get his eyes tested before going to school. Then it will probably be much less. So if the schools tell them that they don't want my child to get an eye certificate before admission or if we want to see a note that the eye has been scanned with the EPI vaccine.Now it is seen that many big centers even counsel if there is any genetic defect. We don't need that. Whether he has a birth defect or not, if a prediction is written that there is no birth defect, then the eyes are also checked. We can detect his squint, ptosis, and character very early.
