# Supplementary material for: Barriers to the uptake of eye health services of the children in rural Bangladesh: A community-based cross-sectional survey
Source: PLoS One. 2023 Dec 7;18(12):e0295341. doi: 10.1371/journal.pone.0295341 (PMC10703229; doi:10.1371/journal.pone.0295341)
Supplement: Attachment — Submitted filename: PONE-D-23-17474-Al-01August2023.pdf [file pone.0295341.s005.pdf]

PLOS ONE

Barriers to the Uptake of Eye Health Services of the Children in rural Bangladesh: A community based cross-sectional survey

--Manuscript Draft--

**Manuscript Number:**	PONE-D-23-17474
**Article Type:**	Research Article
**Full Title:**	Barriers to the Uptake of Eye Health Services of the Children in rural Bangladesh: A community based cross-sectional survey
**Short Title:**	Child ocular health
**Corresponding Author:**	AHM Enayet Hussain<br>Directorate General of Health Services<br>BANGLADESH
**Keywords:**	Children, Eye, Health-seeking behavior, ~~LMICs,~~ Ocular health, Bangladesh
**Abstract:**	Globally, ocular morbidity and disability among children are major public health concerns. This study was designed to explore the health-seeking behaviors of parents in Bangladesh whose children have ocular problems. A cross-sectional mixed method was followed for this study. Method designed to measure eye health care seeking practices of caregivers/parents having children with ocular morbidity in three unions (the lowest administrative geographical area comprised 30,000-50000 population) of the Raiganj Upazila under the Sirajganj District of Bangladesh. The study period was from January to April 2017. Face-to-face interview using a semi-structured ~~quantitative~~ questionnaire with the caregivers were used and KI was conducted among the health service providers during the study period. This was so far the first community based study conducted globally to find out the health seeking behavior of caregivers with identified ocular morbidity. Among 198 confirmed cases of childhood ocular problems, only 87 (43.9%) parents sought health care for their children's ocular morbidities. Better health-seeking behavior was found among the wealthier families. Proportions were 55.3% and 36% among wealthy and low-income families. Affluent families sought care from qualified service providers. Educated household heads chose qualified service providers for their children at a higher rate than illiterate household heads. Lack of knowledge, lack of awareness and financial constraints are significant barriers to seeking proper health care.<br>Conclusion: More than half of the caregivers did not seek any eye care services for their children. Socio-demographic factors, financial constraint play an essential role in the health-seeking behavior of the parents.
**Order of Authors:**	AHM Enayet Hussain<br>Labida Islam, Mph<br>Saidur Rahman Mashreky, MBBS,Mph, PhD<br>A.K.M Fazlur Rahman, MBBS,Mph, PhD<br>Eija Viitasara, PhD<br>Koustuv Dalal, PhD
**Additional Information:**	
**Question**	**Response**
**Financial Disclosure**<br><br>Enter a financial disclosure statement that describes the sources of funding for the work included in this submission. Review the submission guidelines for detailed requirements. View published research	The author(s) received no specific funding for this work.

Powered by Editorial Manager® and ProduXion Manager® from Aries Systems Corporation

articles from *PLOS ONE* for specific examples.<br><br>This statement is required for submission and **will appear in the published article** if the submission is accepted. Please make sure it is accurate.<br><br>**Unfunded studies**<br>Enter: *The author(s) received no specific funding for this work.*<br><br>**Funded studies**<br>Enter a statement with the following details:<br>• Initials of the authors who received each award<br>• Grant numbers awarded to each author<br>• The full name of each funder<br>• URL of each funder website<br>• Did the sponsors or funders play any role in the study design, data collection and analysis, decision to publish, or preparation of the manuscript?<br>• **NO** - Include this sentence at the end of your statement: *The funders had no role in study design, data collection and analysis, decision to publish, or preparation of the manuscript.*<br>• **YES** - Specify the role(s) played.<br><br>* typeset	
**Competing Interests**<br><br>Use the instructions below to enter a competing interest statement for this submission. On behalf of all authors, disclose any competing interests that could be perceived to bias this work—acknowledging all financial support and any other relevant financial or non-financial competing interests.<br><br>This statement is required for submission and **will appear in the published article** if the submission is accepted. Please make sure it is accurate and that any funding sources listed in your Funding Information later in the submission form are also declared in your Financial Disclosure statement.	he authors have declared that no competing interests exist.

View published research articles from [*PLOS ONE*] for specific examples.<br><br>**NO authors have competing interests**<br><br>Enter: *The authors have declared that no competing interests exist.*<br><br>**Authors with competing interests**<br><br>Enter competing interest details beginning with this statement:<br><br>*I have read the journal's policy and the authors of this manuscript have the following competing interests: [insert competing interests here]*<br><br>* typeset	
**Ethics Statement**<br><br>Enter an ethics statement for this submission. This statement is required if the study involved:<br><br>• Human participants<br>• Human specimens or tissue<br>• Vertebrate animals or cephalopods<br>• Vertebrate embryos or tissues<br>• Field research<br><br>Write "N/A" if the submission does not require an ethics statement.<br><br>General guidance is provided below. Consult the submission guidelines for detailed instructions. **Make sure that all information entered here is included in the Methods section of the manuscript.**	Memo No.: CIPRB/ERC/2016/006<br><br>With reference to your application on the above subject, this is to inform you that your research<br>proposal titled "Developing a Framework for Prevention of Childhood Blindness in a Low and<br>Middle Income Country Perspective: Epidemiological Appraisals" has been reviewed and<br>approved by the Ethical Review Committee of the Centre for Injury Prevention and Research, .""",<br>Bangladesh (CIPRB).

Powered by Editorial Manager® and ProduXion Manager® from Aries Systems Corporation

**Format for specific study types**<br><br>**Human Subject Research (involving human participants and/or tissue)**<br>• Give the name of the institutional review board or ethics committee that approved the study<br>• Include the approval number and/or a statement indicating approval of this research<br>• Indicate the form of consent obtained (written/oral) or the reason that consent was not obtained (e.g. the data were analyzed anonymously)<br><br>**Animal Research (involving vertebrate animals, embryos or tissues)**<br>• Provide the name of the Institutional Animal Care and Use Committee (IACUC) or other relevant ethics board that reviewed the study protocol, and indicate whether they approved this research or granted a formal waiver of ethical approval<br>• Include an approval number if one was obtained<br>• If the study involved *non-human primates*, add *additional details* about animal welfare and steps taken to ameliorate suffering<br>• If anesthesia, euthanasia, or any kind of animal sacrifice is part of the study, include briefly which substances and/or methods were applied<br><br>**Field Research**<br><br>Include the following details if this study involves the collection of plant, animal, or other materials from a natural setting:<br>• Field permit number<br>• Name of the institution or relevant body that granted permission	
**Data Availability**<br><br>Authors are required to make all data underlying the findings described fully available, without restriction, and from the time of publication. PLOS allows rare exceptions to address legal and ethical concerns. See the PLOS Data Policy and FAQ for detailed information.	Yes - all data are fully available without restriction

Powered by Editorial Manager® and ProduXion Manager® from Aries Systems Corporation

A Data Availability Statement describing where the data can be found is required at submission. Your answers to this question constitute the Data Availability Statement and **will be published in the article**, if accepted.<br><br>**Important:** Stating 'data available on request from the author' is not sufficient. If your data are only available upon request, select 'No' for the first question and explain your exceptional situation in the text box.<br><br>Do the authors confirm that all data underlying the findings described in their manuscript are fully available without restriction?	
**Describe where the data may be found in full sentences. If you are copying our sample text, replace any instances of XXX with the appropriate details.**<br><br>• If the data are **held or will be held in a public repository**, include URLs, accession numbers or DOIs. If this information will only be available after acceptance, indicate this by ticking the box below. For example: *All XXX files are available from the XXX database (accession number(s) XXX, XXX.)*.<br>• If the data are all contained **within the manuscript and/or Supporting Information files**, enter the following: *All relevant data are within the manuscript and its Supporting Information files.*<br>• If neither of these applies but you are able to provide **details of access elsewhere**, with or without limitations, please do so. For example:<br><br>*Data cannot be shared publicly because of [XXX]. Data are available from the XXX Institutional Data Access / Ethics Committee (contact via XXX) for researchers who meet the criteria for access to confidential data.*<br><br>*The data underlying the results presented in the study are available from (include the name of the third party*	All relevant data are within the manuscript and its Supporting Information files.

and contact information or URL).<br>• This text is appropriate if the data are owned by a third party and authors do not have permission to share the data.<br><br>* typeset	
Additional data availability information:	

 Click here to access/download;Manuscript;6.4.23 PLOS OCULAR HEALTH SEEKING BANGLADESH PLOS.docx

RESEARCH Article

Barriers to the Uptake of Eye Health Servicesof the Children in rural Bangladesh: A community based cross-sectional survey

AHM Enayet Hussain[1]*, Labida Islam[2,3], Saidur Rahman Mashreky[2,3], A.K.M Fazlur Rahman[2,3], EijaViitasara[1], KoustuvDalal[1]

Short title: Child ocular health (4137 words)

[1]Division of Public Health Science, Department of Health Sciences, Mid Sweden University, Sundsvall, Sweden

[2] Centre for Injury Prevention and Research Bangladesh, Dhaka, Bangladesh,

[3] Bangladesh University of Health Sciences, Dhaka, Bangladesh,

Corresponding author:

Email: enayet.hussain@miun.se

ABSTRACT

Globally, ocular morbidity and disability among children are major public health concerns. This study was designed to explore the health-seeking behaviours of parents in Bangladesh whose children have ocular problems. A cross-sectional mixed method was followed for this study. Method designed to measure eye health care seeking practices of caregivers/parents having children with ocular morbidity in three unions (the lowest administrative geographical area comprised 30,000-50000 population) of the Raiganj Upazila under the Sirajganj District of Bangladesh. The study period was from January to April 2017. Face-to-face interview using a semi-structured quantitative questionnaire with the caregivers were used and KI was conducted among the health service providers during the study period. This was so far the first community based study conducted globally to find out the health seeking behaviour of caregivers with identified ocular morbidity. Among 198 confirmed cases of childhood ocular

problems, only 87 (43.9%) parents sought health care for their children’s ocular morbidities. Better health-seeking behaviour was found among the wealthier families. Proportions were 55.3% and 36% among wealthy and low-income families. Affluent families sought care from qualified service providers. Educated household heads chose qualified service providers for their children at a higher rate than illiterate household heads. Lack of knowledge, lack of awareness and financial constraints are significant barriers to seeking proper health care.

Conclusion: More than half of the caregivers did not seek any eye care services for their children. Socio-demographic factors, financial constraint play an essential role in the health-seeking behaviour of the parents.

Introduction

Globally, ocular morbidity and disability among children are major public health concerns. Infancy and childhood are critical periods for visual development. When problems arise, children may not be able to complain or convey their difficulties, and caregivers may not appreciate or understand the potential for children's visual problems. Delays in interventions may lead to mild to severe permanent visual impairments.

Globally, around 1.4 million children aged 0-14 years live with blindness, while about 17.5 million are at risk for low vision[1]. However, most of these children with visual impairments live in low and middle-income countries with a prevalence of 1.5 per 1000 children, compared with 0.3 per 1000 in high-income countries[2]. Data shows that an estimated 70 million blind-person years are caused by blindness among children[3]. The recent studies in Indiaindicated that more than 30% of the blind population lost their vision before the age of 17 years and, unexpectedly, many of them lost their sight when aged less than five years[4,5]. In low and middle-income countries, optimal eye care is not always available and there are excessively high numbers of cases of avoidable blindness. Globally, 90% of people with blindness live in LMIC, with the most affected disadvantaged and vulnerable communities.

This reflects the link between eye health problems, poverty, and low education[6]. Proper health-seeking behaviour is essential for better health outcomes. Inappropriate health-seeking behaviour can influence worse health outcomes to increase morbidity and mortality [7]. There are significant barriers to seeking eye care services for children. These include lack of availability of eye care services, lack of awareness about the need and availability of services, financial constraints, utilization of time to receive such services and improper referral by general practitioners and paediatricians[8–10].A belief in traditional medicine and taboo has also been barriers against seeking eye care services[8,11]. Knowing eye disease, people neglect to seek eye care services and there exists a large gap between mass awareness and treatment practices about common eye diseases in low and middle-income countries[12,13].

Childhood blindness and visual impairments constitute a significant proportion of ocular morbidities in developing countries which can be treated and prevented with strategic efforts[14]. The prevention of ocular morbidities among children requires early diagnosis and treatment. Two different studies conducted in Chennai, India and Nigeria indicated that seeking eye care for children depends on parental awareness and perception about their children's eye health[15,16].

Additionally, demographic factors affect the utilization of eye care services such as age, race, gender, socioeconomic status, education, knowledge about eye diseases, and services available for eye care[17].

Like in other low and middle-income countries, ocular morbidity is an established public health problem in Bangladesh. Relevant recent epidemiological data on the burden of this existing problem is scarce. A study showed that the prevalence of childhood blindness was 6.3 per 10,000 among children aged $\leq$ 15 years[18]. There are limited population-based studies considering both the blind and non-blind causes of childhood visual problems. Moreover, almost no data have been found on the health-seeking behavioural pattern of the

caregivers/ parents towards childhood ocular problems. This study was designed to explore the health-seeking behaviours of parents in Bangladesh whose children have ocular problems. The findings of this study was facilitated the development of a national strategy for attaining better health-seeking behaviours of parents to reduce the consequences of childhood visual problems.

Materials and Methods

It was a cross-sectional mixed method study. Both qualitative and quantitative methods were used to explore the eye health care-seeking practices among the caregivers/parents having children with ocular morbidity in three unions (the lowest administrative geographical area comprised 30,000-50000 population) of the Raiganj Upazila, under the Sirajganj District of Bangladesh. The study period was from January to April 2017. The details of the study methods, including study population and data collection procedures, have been described elsewhere.[19]

Patient and Public involvement: Both patients' care giver and service provider interviewed in this study. Initially, a quantitative face-to-face interview was conducted by trained field workers (3 field supervisors and 15 data collectors) to identify the children with visual problems. The data collectors identified five hundred and seventy cases among 39,351 children at the field level. All cases were invited to join another session organized into the local office of CIPRB. A team of 5 paediatric ophthalmologists then confirmed the screened-out cases. The ophthalmologist kept the record of the principal reason and the contributing reasons for the children's visual impairments and blindness. In total, 198 cases were confirmed by the team as children having ocular morbidity.

The next stage parents or caregiver of 198 patients were interviewed to explore their health seeking behavior for children with ocular illness. Face-to-face interviews were conducted

using a structured questionnaire. A team of 2 trained data collectors conducted the interviews. In total, 198 caregivers participated in the study. Socio-demographic information of the respondents was collected from the database of the CIPRB surveillance system. All individuals in the surveillance area have ~~their~~ unique identification numbers. After collecting specific information, it was merged with the required socio-demographic variables of the existing population database.

Researcher himself with a research assistant conducted the qualitative interviews. A total of 6 Key Informant Interview (KII) was conducted with different level of service providers whom are Pediatric Ophthalmologist. Conducting interview with the caregivers, data collectors conducted KIIs with the Pediatric Ophthalmologist from tertiary hospital and primary care center. In total 6 KIIs were conducted. All KIIs were electronically audio-recorded and hand notes taken where appropriate.

Data analysis

A descriptive analysis was carried out to describe the population characteristics both for the children and the households. In this analysis, we considered the age and gender of the children and age, literacy, occupation, and socioeconomic status of the head of households independent variables. The type of service provider was considered dependent variable Results were shown as percentages according to the type of service provider. A Chi-square test was done to see the statistical significance between dependent and independent variables. $P < 0.05$ was considered statistically significant. Seeking service and seeking service from qualified service providers were the dependent variables. Age and sex of the children, age, education and occupation of the household head, and household economic condition were considered independent viable. In qualitative part all audio-recording was transcribed and from them result was zest out. Thematic analysis was carried out for the qualitative part.

Analysis of qualitative data

The discussions were transcribed very carefully and accurately by two independent research assistants and then compared for accuracy. The transcriptions were then coded independently by the two research assistants. By content analysis major themes were identified, coded, and categorized. Validity was ensured by comparing the researchers' findings among themselves and with those of an independent investigator. No major inter-rater inconsistencies were found. After sorting and categorizing the responses, excerpts from the transcripts were chosen to illustrate the summary statements, which were also used to validate the findings. Transcription was made in Bangla then it was translated into English.

Operational Definitions

Ocular morbidities: This encompassed childhood blinding and non-blinding conditions. The definition used for childhood blindness is described elsewhere [19].

Qualified service providers: Public and private hospitals, clinics and health facilities, and private registered physicians.

Non-qualified service providers: These included government and NGO's field-level health workers, village doctors, drug sellers, herbal/homoeopathic practitioners, traditional/ religious healers and others.

Wealth index: The definition of the wealth index used for this study has already been published elsewhere[20].

Literacy of household head: A household head who attended no school education, informal education and only one year of schooling was considered 'No Education'. A primary level of education was defined as when the household head had five completed years of schooling. Those who attended more than five years of school education were designated as 'SSC and above'.

Occupation of household head: The unskilled worker group included those involved in house duties, unskilled labour, servant and retired, whereas the skilled worker group included the categories of service holder and skilled labourer.

Ethical Consideration

Ethical clearance for this study was received from the Ethical Review Committee of the Centre for Injury Prevention and Research, Bangladesh (CIPRB/ERC/2016/006). Written consent of all caregivers was gained before conducting the study.

RESULTS

In this study, the proportion of male children (51.4%) is quite similar to that of female children (48.6%). The majority of the children (70.0%) were between 5 and 15 years of age. About two-thirds of the children's household heads were in the young age group (64.7%). Most of the household heads were illiterate (61.6%) and belonged to the poor socioeconomic condition (50.0%), as shown in (Table 1).

Table 1: Population characteristics (N=39351)

Variables	Frequency	Percentage
**Children's characteristics**		
**Sex of the child**		
Female	19140	48.6
Male	20211	51.4
**Age of the child (in years)**		
5 to 15 years	27545	70.0

<5 years	11806	30.0
**Literacy of household head**		
No education	24243	61.6
Primary	11856	30.1
SSC and above	3252	8.3
**Age of household head**		
<35yrs	25462	64.7
36-59	10968	27.9
60 and above	2921	7.4
**Occupation of household head**		
Agriculture	10254	26.1
Unskilled worker	15005	38.1
Skilled worker	9163	23.3
Business	4929	12.5
**Wealth index**		
Poor	19678	50.0
Middle class	9843	25.0
Rich	9830	25.0

A total of198 confirmed cases of childhood ocular morbidities, including blindness, were identified; among them, only 87 (43.9%) were sought health care for their ocular morbidities—those who sought health care more than one third sought non-qualified health

care providers. Among the 43.9%, around 14% of providers were herbal/homoeopathic practitioners and about 5% were medicine shopkeepers(Fig 1 and Fig 2).

Figure 1: Health seeking pattern for childhood ocular morbidities in a rural community of Bangladesh.

Figure 2: Health seeking pattern for childhood eye problems in rural Bangladesh

Health seeking behaviour was found better among the wealthier families. A higher proportion of families in the more affluent group sought health care for their children with an ocular problem. It was 55.3% and 36% among wealthy and low-income families, respectively. A higher proportion of wealthy families sought care from qualified service providers than low-income families. It was 90.5% and 52.5%, respectively. The difference was statistically significant in both of the cases. Similarly, a higher proportion of families sought care for their children when a household is literate than a family with an illiterate household head.
Educated household heads chose qualified service providers for their children at a higher rate than illiterate household heads. Seeking service and choosing a qualified service provider found when a child was male in gender (Table 2).

Table 2: Socio-demographic characteristics and care seeking pattern of childhood eye problem

	**Service received**			**Type of Service received**		
	Yes	No	P	Non-qualified	Qualified	P
**Age of child**						
**5 & Above**	43.7	56.3	0.90	32.3	67.7	0.30

**Under-5**	44.6	55.4		44.0	56.0	
**Gender of child**						
**female**	38.5	61.5	0.09	37.1	62.9	0.49
**male**	48.6	51.4		34.6	65.4	
**Education of HH head**						
**No education**	41.4	58.6	0.14	41.5	58.5	0.35
**Primary**	37.5	62.5		25.0	75.0	
**SSC and above**	57.9	42.1		27.3	72.7	
**Occupation of HH head**						
**Unskilled**	47.8	52.2	0.80	31.3	68.7	0.31
**Agri**	40.3	59.7		44.0	56.0	
**Busi**	40.0	60.0		16.7	83.3	
**Skilled**	46.2	53.8		44.4	55.6	
**Age of HH head**						
**17- 35 yrs**	38.9	61.1	0.13	41.2	58.8	0.13
**36- 59 yrs**	52.1	47.9		36.0	64.0	
**60yrs & above**	57.9	42.1		9.1	90.9	
**Economic condition**						
**Poor**	36.0	64.0	0.04	47.5	52.5	0.01
**Middle**	53.1	46.9		38.5	61.5	
**Rich**	55.3	44.7		9.5	90.5	

The leading barriers responsible for not seeking services were lack of knowledge, social taboo and financial constraints with ~~the~~ proportions of 51.4%, 22.5% and 18.9%, respectively (Fig 3).

Figure 3: Barriers to seeking health care services for childhood blindness in a rural community in Bangladesh. (N=111)

Qualitative Findings

In this section three main themes were carried out by thematic analysis. Theme one was lack of knowledge which reflected their knowledge of eye health care seeking. Theme two and three were lack of awareness and economic constraints which was also reflected in quantitative result.

Lack of Knowledge about the disease and its consequence

All the participants of qualitative part stated that lack of knowledge was the key barriers of eye health care seeking. All of them mentioned that parents do not have adequate knowledge about eye diseases of their children. Due to lack of knowledge of their parents they did not give it any importance when they come to come know any complain about occular illness. In many cases parents did not know where to seek eye care for their children. One the service provider participant stated

"Some parents are aware that their children have some eye problem but they did not know where to seek paediatric eye care".

Lack of awareness about children's ocular illness

Aware or lack of awareness about children's ocular illness was another barrier for seeking eye care. Participants narrated that most of the time parents were ignorant about their children's eye due to proper awareness. Parents thought it will be ok on the children's later age. One of the participants also expressed that

"Child's cannot complain and mothers cannot understand the problem as they were not well aware about paediatric eye problem"

Economic constraints

Qualitative finding revealed economic constraints are one of the barriers for health seeking behaviour. According to the views of the respondents economy was one of the main reasons that rural people did not seek care for their children's eye problem.

A respondent mentioned that "As our out-pocket expenditure was high, so that people from rural area did not seek paediatric eye care as they had to pay lot of money for transportation as well as for treatment purpose".

DISCUSSION

This study was designed to explore the health-seeking behaviour of the parent for their children with ocular morbidities and disabilities. This study was conducted among the parents who had a child with ocular morbidity and service providers from primary and tertiary level eye care. We explored the health-seeking behaviour of the parents of those 198 children having ocular problem and the service provider who are giving eye care at primary and tertiary care level.

In this study more than half the parents did not seek health care for their children with ocular morbidities. A similar finding was observed in a study conducted in Nigeria[21]. A national survey found that parents sought health care at a lower rate for common illnesses like common cold and diarrhoea[22]. However, better health-seeking behaviour was found in a study conducted in an urban slum in Dhaka[23]. In our study association was found between the health-seeking behaviour of the parents and their economic condition educational qualification. Parents seek health care for their children at a higher rate when their economic condition improves. The wealthy family also chose qualified health care providers for their children. In Bangladesh, a similar association was observed to health-seeking behaviour for their children in other health-related events[24]. A study in India shows that lack of money is one of the significant barriers to seeking eye care for their children[25]. Also in this study,

economic constrains is the major barrier for seeking eye care result found from the qualitative part. Study conducted in Nigeria supported the same result[26]. Eeducated parents who sought health care for their children at a higher rate. They also choose qualified health care providers to get care for their children. In the qualitative exploration of this study service provider mentioned lack of knowledge about the disease is a major barrier in seeking health care. A similar finding was observed in the health-seeking of parents for childhood burn injuries in Bangladesh[24]. The finding was consistent with other studies in similar settings[23,27,28]. Compared to parents with younger children, parents sought health care at a higher rate who have children aged five years or more. It might be higher age children can complain about their problem, or symptoms become severe with the progression of ages. On the other hand, lower age group children cannot complain, and their issues remain unaddressed. However, early detection and treatment are essential for a better outcome of an illness. Some interventions can be taken to make parents aware of early age symptoms. We found boys are more prevalent for getting health care and care from quality healthcare providers than girls, although the finding was not statistically significant. A study in Africa found that the care-seeking pattern is strongly related to the perceived severity of the illness[11].

There are many possible psychosocial and economic barriers to seeking eye care services for children. For example, it has been shown from studies in India and Africa that financial constraints are a major barrier for parents to seek eye care services for their children [11,25]. In this study, caregivers' lack of knowledge, lack of awareness, financial constraints, and social taboo about childhood ocular problems were deemed the major barrier to seeking eye health services in Bangladesh. Studies conducted in other countries about parents' awareness and perceptions of childhood ocular problems have also illustrated that parents' knowledge and understanding of the aetiology of eye health problems can be lacking. Such factors may

influence caregivers' healthcare behaviours and militate against seeking help for their children [16].

More than half of the parents did not seek eye care services for their children. Of those who sought health care, one-third sought care from an unqualified service provider. Socio-demographic factors, including lack of awareness, play an important role in seeking eye care services. A comprehensive strategy needs to be developed to address childhood ocular morbidities, including childhood blindness. Two significant factors must be addressed. There need to be comprehensive screening facilities available at the community level to facilitate the early detection of eye health problems. Additionally, caregivers must become more aware of possible eye health problems and the merits of early interventions when problems occur.

Strength and limitation

This was so far the first community based study conducted globally to find out the health seeking behaviour of caregivers/parents of the child who have ocular problem. Our findings add significant impact for preventing childhood ocular problems. Policy makers can utilize this knowledge to formulate an action plan to reduce childhood blindness by integrating intra and inter-sectoral intervention. Though this is the first community based study and this study conducted in only one sub-district, thus this is not a national representative study.

Conclusion

More than half of the caregivers did not seek any eye care services for their children. Socio-demographic factors, including lack of awareness, play an essential role in the health-seeking behaviour of the parents. Improved healthcare-seeking behaviours may be facilitated by improving caregivers' knowledge and attitudes towards eye health problems and how problems may be addressed. There will be increased opportunities to reduce childhood ocular morbidity and disability and prevent unnecessary visual problems and blindness in low resource settings in developing countries.

Acknowledgement

This study was conducted in the surveillance area of the Centre for Injury Prevention and Research Bangladesh (CIPRB). The CIPRB provided administrative and technical support in the data collection and data management procedures. We are also grateful to the ophthalmologists who took part in the visits for the detection of the confirmed cases. We are thankful to the members of the office of the Director General of Health Services for their technical support in the validation of our research materials.

Completing Interest

The authors have declared that no competing interests exist.

Authors Contribution

All the authors wrote, read final version and approved the paper.

Enayet Hussain: Conceptualize, literature support, data collection, analysis, review and writing-original draft.

Labida Islam: Conceptualize, analysis,writing-original draft

Saidur Rahman Mashreky: conceptualize, analysis, writing and critical review.

A.K.M Fazlur Rahman: writing and critical review

Eija Viitasara: writing and critical review

Koustuv Dalal: Conceptualize, planning, analysis, writing, critical review and overall supervision.

REFERENCES

1. WHO. Global Data on. Glob Data Vis Impair 2010. 2010;17.
2. Gilbert CE, Anderton L, Dandona L, Foster A. Prevalence of visual impairment in children: a review of available data. Ophthalmic Epidemiol [Internet]. 1999 Mar 1 [cited 2022 Sep 24];6(1):73–82. Available from: https://europepmc.org/article/med/10384686
3. Yorston D, Naughton A. Community. 2020;
4. Gupta M, Gupta BP, Chauhan A, Bhardwaj A. Ocular morbidity prevalence among school children in Shimla, Himachal, North India. Indian J Ophthalmol. 2009;57(2):133–8.
5. Faisalabad CIN. VISUAL SCREENING ; 2020;112–6.
6. Pascolini D, Mariotti SP. Global estimates of visual impairment: 2010. Br J Ophthalmol [Internet]. 2012 May [cited 2022 Oct 22];96(5):614–8. Available from: https://pubmed.ncbi.nlm.nih.gov/22133988/
7. Latunji OO, Akinyemi OO. FACTORS INFLUENCING HEALTH-SEEKING BEHAVIOUR AMONG CIVIL SERVANTS IN IBADAN, NIGERIA. Ann Ibadan Postgrad Med. 2018;16(1):52–60.
8. Balasubramaniam SM, Kumar DS, Kumaran SE, Ramani KK. Factors affecting eye care-seeking behavior of parents for their children. Optom Vis Sci. 2013;90(10):1138–42.
9. Akowuah PK. Barriers to Utilization of Eye Care Services in the Upper East Region, Ghana. Adv Ophthalmol Vis Syst. 2017;7(6):405–7.
10. Fletcher AE. Low Uptake of Eye Services in Rural India. Arch Ophthalmol. 1999;117(10):1393.
11. Ebeigbe JA. Factors influencing eye-care seeking behaviour of parents for their

children in Nigeria. Clin Exp Optom. 2018;101(4):560–4.

12. Ormsby GM, Arnold AL, Busija L, Mörchen M, Bonn TS, Keeffe JE. The Impact of Knowledge and Attitudes on Access to Eye-Care Services in Cambodia. Asia-Pacific J Ophthalmol. 2012;

13. Islam FMA, Chakrabarti R, Islam SZ, Finger RP, Critchley C. Factors associated with awareness, attitudes and practices regarding common eye diseases in the general population in a rural district in Bangladesh: The Bangladesh population-based diabetes and eye study (BPDES). PLoS One. 2015;10(7).

14. Pararajasegaram R. The global initiative for the elimination of avoidable blindness. Community Eye Heal J. 1998;11(26):29.

15. Senthilkumar D, Balasubramaniam S, Kumaran SE, Ramani K. Parents’ Awareness and Perception of Children’s Eye Diseases in Chennai, India. 2013;(December).

16. Ebeigbe JA, Emedike CM. Parents ’ awareness and perception of children ’ s eye diseases in Nigeria. J Optom. 2017;10(2):104–10.

17. Park YS, Heo H, Ye BJ, Suh Y woo, Kim S hyun, Park SH. Prevalence and Factors Associated with the Use of Eye Care Services in South Korea : Korea National Health and Nutrition Examination Survey 2010 – 2012. 2017;31(1):58–70.

18. Rahman F, Ferdausi N, Dalal K. Epidemiology of childhood blindness : A community-based study in Bangladesh. 2019;1–10.

19. Hussain AHME, Ferdoush J, Mashreky SR, Rahman AKMF, Ferdausi N, Dalal K. Epidemiology of childhood blindness: A community-based study in Bangladesh. PLoS One [Internet]. 2019 Jun 1 [cited 2021 Nov 1];14(6):e0211991. Available from: https://journals.plos.org/plosone/article?id=10.1371/journal.pone.0211991

20. Rutstein SO, Johnson K. The DHS Wealth Index. DHS Comp Reports No 6. 2004;(December):1–71.

21. Senyonjo L, Lindfield R, Mahmoud A, Kahaki K, Sanda S, Schmidt E. Ocular Morbidity and Health Seeking Behaviour in KwaraState, Nigeria: Implications for Delivery of Eye CareServices. PLoS One. 2014;9(8).

22. Sabbir Ahmed M, Yunus FM. Healthcare seeking behavior for common illness among Bangladeshi under-five children: a nationwide cross-sectional survey. Child Youth Serv Rev. 2020 Dec 1;119.

23. Mahejabin F, Parveen S, Ibrahim M. Mother's / care giver's health seeking behaviour during childhood illness in an urban slum of Dhaka city. Pulse. 2015;7(1):5–15.

24. Mashreky SR, Rahman A, Chowdhury SM, Svanströ L, Shafinaz S, Khan TF, et al. Health seeking behaviour of parents of burned children in Bangladesh is related to family socioeconomics.

25. Balasubramaniam SM, Kumar DS, Evangeline KS, Ramani KK. Factors Affecting Eye Care Y Seeking Behavior of Parents for Their Children. Optom Vis Sci. 2013;90(10):1138–42.

26. Ebeigbe JA. Factors influencing eye- care seeking behaviour of parents for their children in Nigeria. https://doi.org/101111/cxo12506 [Internet]. 2021 Jul 1 [cited 2023 Jan 19];101(4):560–4. Available from: https://www.tandfonline.com/doi/abs/10.1111/cxo.12506

27. Apuleni G, Jacobs C, Musonda P. Predictors of Health Seeking Behaviours for Common Childhood Illnesses in Poor Resource Settings in Zambia, A Community Cross Sectional Study. Front Public Heal. 2021;9(May):1–9.

28. Chandwani H, Pandor J. Healthcare-Seeking Behaviors of Mothers regarding their Children in a Tribal Community of Gujarat, India. Electron physician. 2015;7(1):990–7.

Figure 1

Click here to access/download;Figure;Fig 1.tif

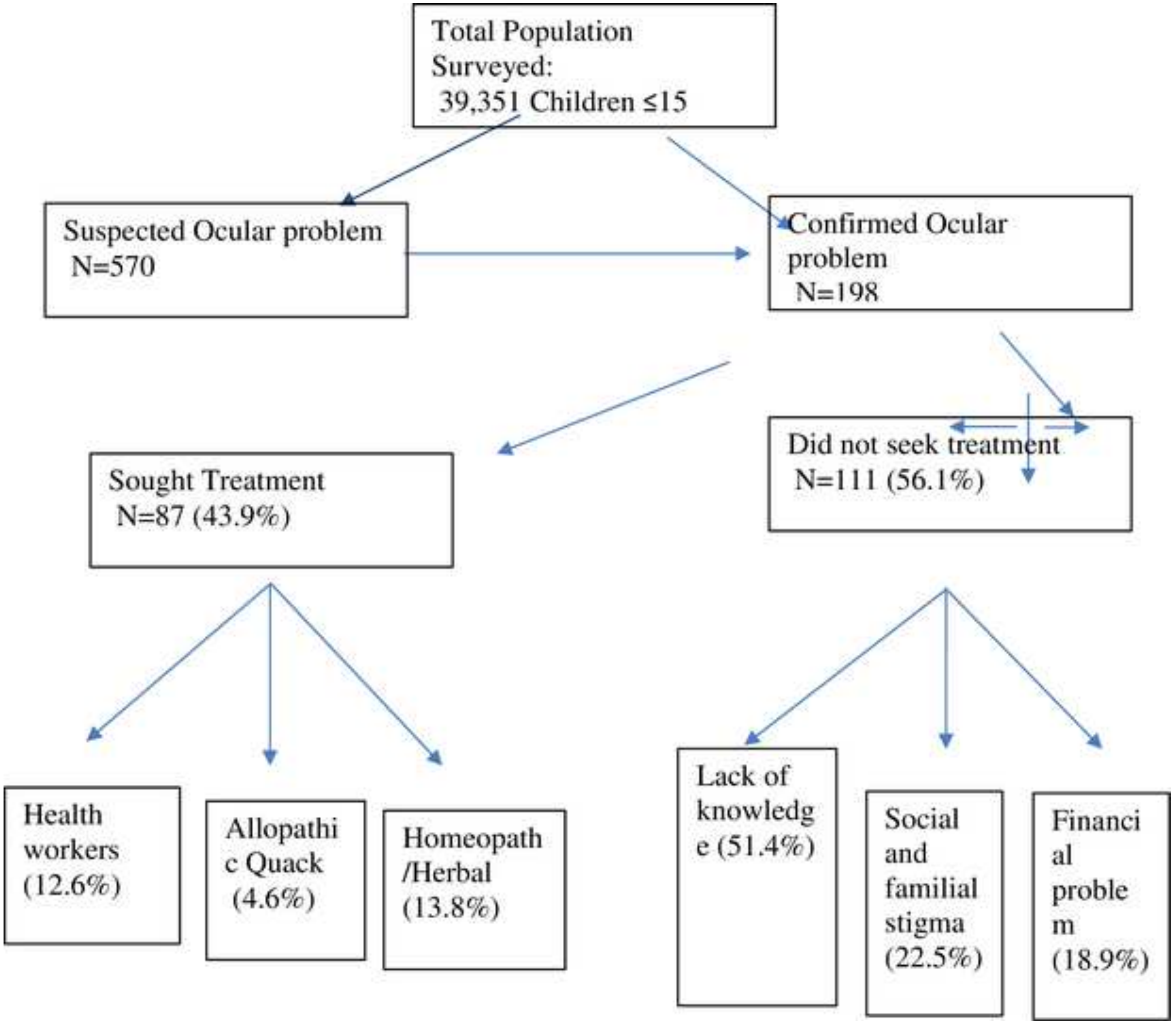

Figure 2

Click here to access/download;Figure;Fig 2.tif

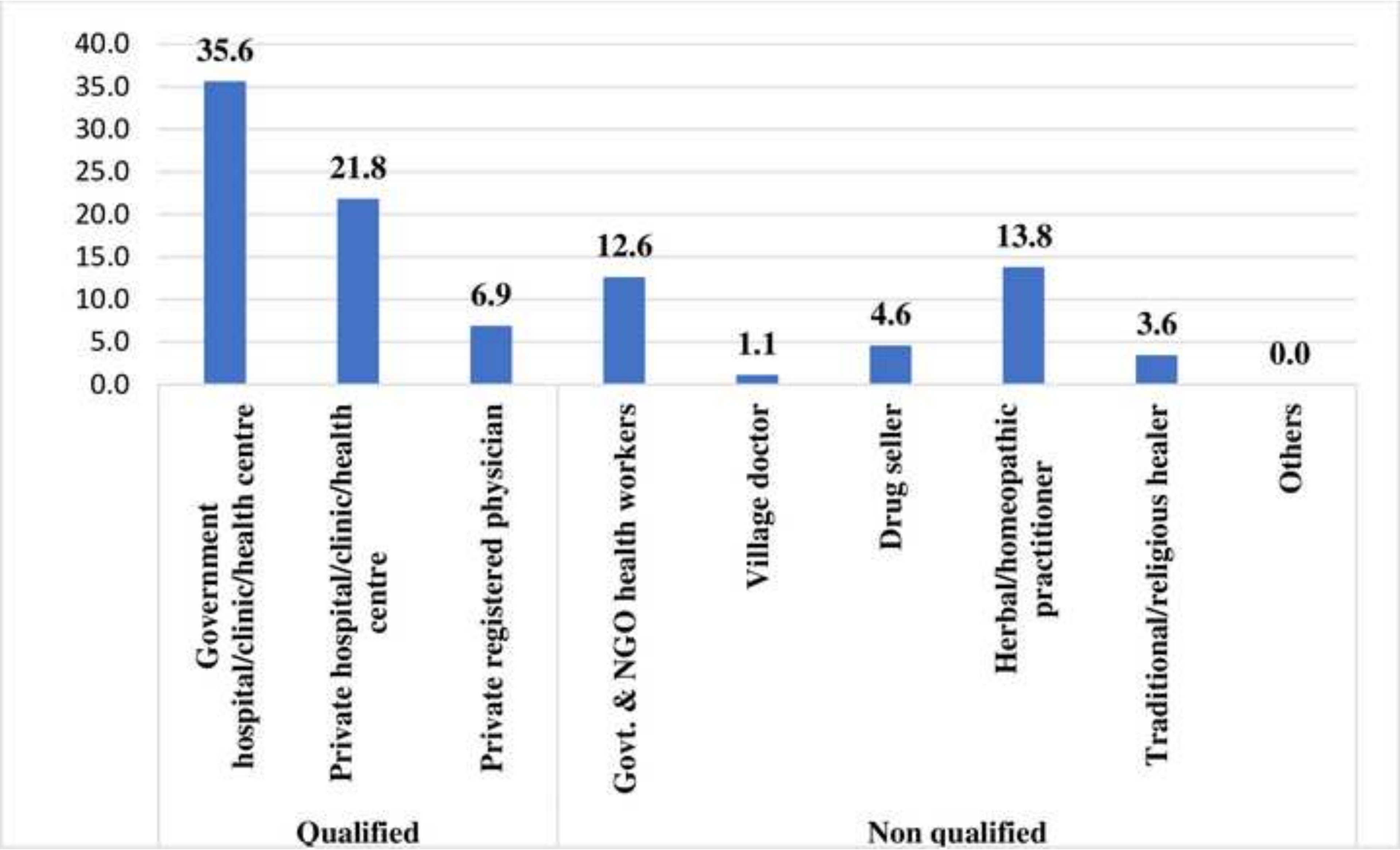

Figure 3

Click here to access/download;Figure;Fig 3.tif

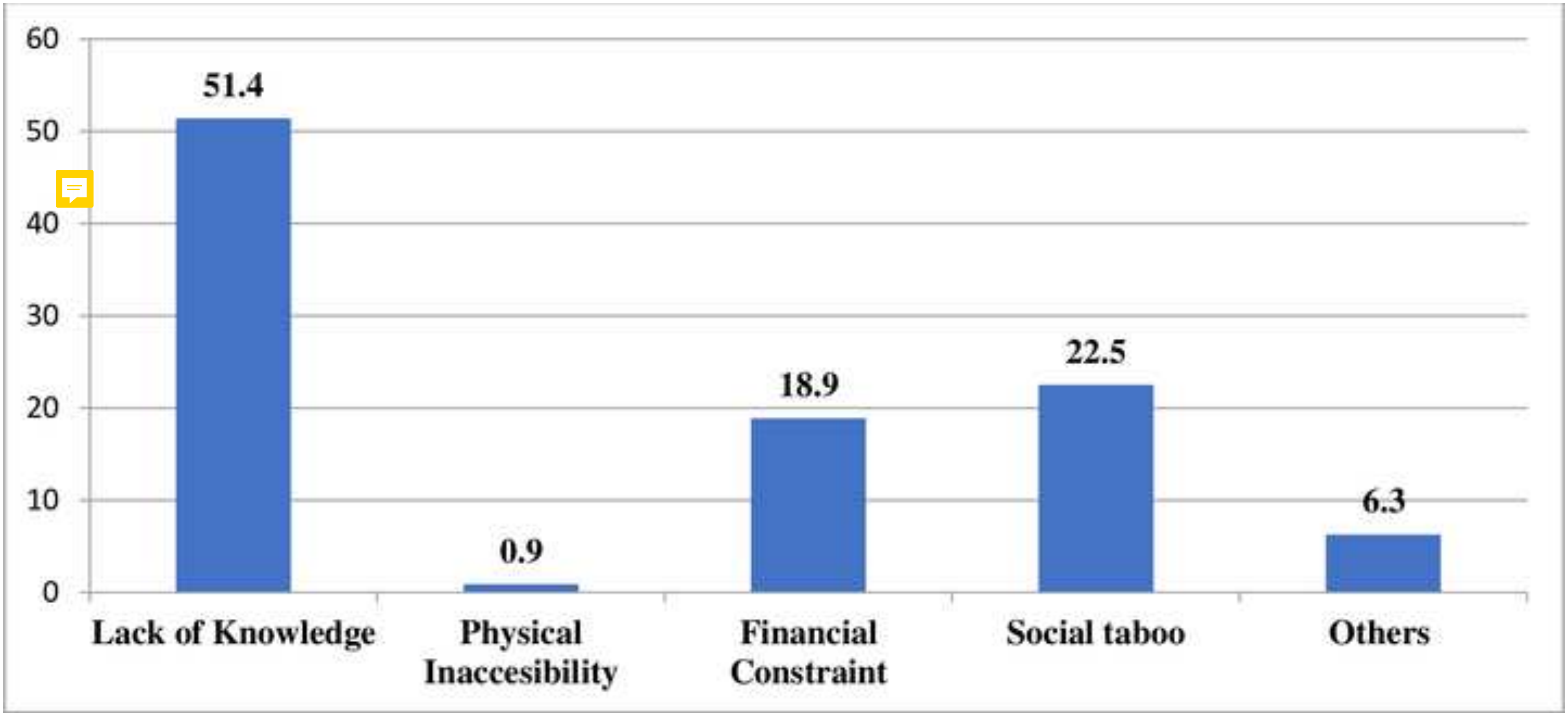

Click here to access/download
Supporting Information
STROBE-checklist-v4-combined.doc
