# Supplementary material for: Barriers to the uptake of eye health services of the children in rural Bangladesh: A community-based cross-sectional survey
Source: PLoS One. 2023 Dec 7;18(12):e0295341. doi: 10.1371/journal.pone.0295341 (PMC10703229; doi:10.1371/journal.pone.0295341)
Supplement: Attachment — Submitted filename: Resonse to Reviewers.docx [file pone.0295341.s006.docx]

To

The Editors

PLOS ONE

Dear Editors

Thank you for sending the comments on our manuscript titled “Barriers to the Uptake of Eye Health Servicesof the Children in rural Bangladesh: A community based cross-sectional survey”. On behalf of my team, I would also like to thank you and the reviewers for your insightful comments, which will help us improve our article. Please locate the revised drafts and the comments' corresponding responses in the following page.

Thank you.

Your Sincerely

A.H.M Enayet Hussain

| **Academic Editor** | Thank you for your positive feedback. As per your suggestion Data file uploaded as a supporting information. |
| --- | --- |

| **Reviewer 1** | |
| --- | --- |
| Reference checking could not be completed due to a system error. Please check and/or verify again. | Thank you for your valuable feedback. We have revised the reference issues in the manuscript and the changes are in the following line number 44, 358 and 366. |
| I have a few queries and/or comments. Please make the necessary corrections. | Thank you once again. Corrections are made in the following document below as well as in the manuscript with track change. |

| **Reviewer 2** | |
| --- | --- |
| How did the author categorize the respondent’s occupation? Skilled and unskilled is not a part of occupation. Author can consider them as wage workers. | Thank you for your comment in the manuscripts. Revision was made in the manuscripts in both table 1 and 2. Changes made in the line number 176 and 200. And an operational definition was described in the operational definition section as “Skilled worker refers to a job where s/he require judgement to perform the assigned duties including service sectors. The unskilled worker refers to job requiring unimportant or no judgement to perform the assigned duties including household works, untrained work without any skills” in the line number 161 to 164. |
| Explain about non-qualified and qualified services. how can a reader differentiate them? | Thank you for your response. As per the operational definition of the manuscripts; Public and private hospitals, clinics and health facilities, and private registered physicians are defined as qualified service providers in this paper.  And government and NGO's field-level health workers, village doctors, drug sellers, herbal/homoeopathic practitioners, traditional/ religious healers and others are defined as non- qualified service providers  Operational definition of this two was described in the 146 and 148 no line in the manuscript. |
| The qualitative findings do not follow the proposed analysis methods. Author can quote the field narratives for depth understanding of the issues. | Thank you for this comment. Revision was made according to your comments in the line number 134 to 142 and 211 to 249. |
| what are the government intervention to mitigate the issue. What is the state mechanism promoting awareness and knowledge about children's ocular illness. Author should discuss their policy and programme for finding the gaps. | Bangladesh has successfully integrated primary eye health care for children into the country's Integrated Management of Childhood Illness (IMCI) programme in 2018, where identifying eye problems and strong referral mechanisms to the eye department at district hospital were developed. Community health workers attached to facilities delivering IMCI were also engaged to promote awareness about eye conditions in children in the community.    The findings of this study will contribute to the formulation of effective policies aimed at enhancing health-seeking behaviour, particularly concerning the ocular health of children in Bangladesh.  This section was discussed in the discussion section in the line number 308 to 313. |

| **Other comments in the pdf file by Authors** | |
| --- | --- |
| Thank you for all the comments in our manuscript. Necessary changes are listed below according to your comments. | |
| Title | Revision was made according to your suggestion. Correction was done in the line number 2 & 3. Title: **Barriers to the uptake of eye health services of the children in rural Bangladesh: A community based cross-sectional survey** |
| Abstract | Necessary changes were made in the line number 19 and 32 to 35. |
| Line number 5: KoustuvDalal | Space was given in between the name in the line number 5. |
| References | Uniformity of the references were made in the document. |
| 56 no line | Space was given before A in the manuscripts in the line number 63. |
| 88 no line | Revision was made according to your suggestion. Caregiver was used in the whole manuscripts in the line number of 90, 96, 106 and 320. |
| Face-to-face interviews were conducted using a structured questionnaire. | Semi-structured questionnaire was used in the manuscript according to your comments in the line number 108. |
| Analysis of qualitative data | Revision was made in the manuscripts in the line number 134 to 142 and 211 to 249.. |
| Table 1 and table 2 | In table 1 and 2 sex, age and occupation were revised accordingly.  Table 1 and 2 were in the line number 176 and 200. |
| 198 no line | Small letter k was used in the line number 216. |
| 199 no line | Rephrasing was done in the line number 217 to 218. |
| 208 no line | Lack of awareness was used in the line number 232. |
| Discussion | Discussion was thoroughly checked for grammatical error and revised in the line number 251 to 316. |
| Strength and limitation | Strength and limitation were revised according to your comments in the line number 318 to 325. |
| 283 no line | In the conclusion a was given before lack of awareness in the line number 328. |
| Figure 3 | Title was given and axis was changes according to your suggestion. |
