# Supplementary material for: Barriers to the uptake of eye health services of the children in rural Bangladesh: A community-based cross-sectional survey
Source: PLoS One. 2023 Dec 7;18(12):e0295341. doi: 10.1371/journal.pone.0295341 (PMC10703229; doi:10.1371/journal.pone.0295341)
Supplement: Attachment — Submitted filename: Resonse to Reviewers.docx [file pone.0295341.s007.docx]

To

The Editors

PLOS ONE

Dear Editors

Thank you for sending the comments on our manuscript titled “Barriers to the Uptake of Eye Health Servicesof the Children in rural Bangladesh: A community based cross-sectional survey”. On behalf of my team, I would also like to thank you and the reviewers for your insightful comments, which will help us improve our article. Please locate the revised drafts and the comments' corresponding responses in the following page.

Thank you.

Your Sincerely

A.H.M Enayet Hussain

| **Academic Editor** |  |
| --- | --- |
| Please review your reference list to ensure that it is complete and correct. If you have cited papers that have been retracted, please include the rationale for doing so in the manuscript text, or remove these references and replace them with relevant current references. Any changes to the reference list should be mentioned in the rebuttal letter that accompanies your revised manuscript. If you need to cite a retracted article, indicate the article’s retracted status in the References list and also include a citation and full reference for the retraction notice. | Thank you for your positive feedback. Reference lists are revised according to your recommendation and retracted paper has removed. Changes are made in the line number 379 to 451. |

| **Reviewer** | |
| --- | --- |
| The author needs to work on qualitative parts. The field narrative is still missing in the corrected version. The result section should be follow the proposed methodology. | Thank you for your valuable feedback. We have revised the whole qualitative parts and the changes made in the following line number 87 to 89, 112 to 139, 162 to 165 and 231 to 273. |
